# Being Mad in Early Modern England

**DOI:** 10.3389/fpsyg.2015.01740

**Published:** 2015-11-19

**Authors:** Aleksandar Dimitrijevic

**Affiliations:** ^1^Psychology, University of BelgradeBelgrade, Serbia; ^2^International Psychoanalytic UniversityBerlin, Germany

**Keywords:** history of mental disorders, early modern period, history of melancholy, Shakespeare, Robert Burton

## Abstract

It has become almost a rule that the birth of scientific psychiatry and what we today term clinical psychology took place in the short period between the last decade of the XVIII century and the 1820s. Everything that happened before that period—every description, diagnosis, and therapy—has been considered “pre-scientific,” outdated, in a way worthless. In this paper, however, I am providing the argument that, first, the roots of contemporary psychiatry reach at least to England of the early modern period, and that, second, it may still turn out that in the field of mental health care historical continuities are more numerous and persistent than discontinuities. Thus, I briefly review the most important surviving documents about the treatment of mental disorders in England of Elizabethan and Jacobian period, organizing the argument around the well-known markers: diagnostics and etiology, therapy, organization of the asylum, the public image of the mentally ill.

It has become almost a rule that the birth of scientific psychiatry and what we today term clinical psychology took place in the short period between the last decade of the eighteenth century and the 1820s. The historians either hail Phillipe Pinel's efforts to free Parisian patients/inmates from chains, or they quote from German medical textbooks that have laid fundaments for psychiatry of the twentieth century, in whose shadows clinicians still work today.

More importantly, every description, diagnosis, and therapy that happened before that period has been considered “pre-scientific,” or outdated. As modern medicine is based on natural sciences, its history is not regarded as a source of relevant knowledge, but is relegated among humanistic disciplines. In this paper, however, I am offering an argument that, first, the roots of contemporary psychiatry reach at least to England of the early modern period, and that, second, it may still turn out that in the field of mental health care historical continuities are more numerous and persistent than discontinuities.

In order to achieve that, I shall briefly review documents about the treatment of mental disorders published and used in England especially, but not exclusively, during Elizabethan and Jacobean eras (i.e., 1558-1625)—organizing the argument around the well-known markers: diagnostics and etiology, therapy, organization of the asylum, the public image of the mentally ill… I will review the books that were revolutionary for that period (like *The Discoverie of Witchcraft*), significantly influenced the development of the field (like *A Briefe Discourse of a Disease Called the Suffocation of the Mother*) or still quoted very frequently today (like *The Anatomy of Melancholie*). I will also add illustrations from Shakespeare's plays, universally considered very accurate and influential (Simpson, 1959; Edgar, 1970; Kail, 1986; Adams, 1989; Iyengar, 2014).

One may well argue that the modern treatment of the mentally ill started when the practice of exorcism used during the Christian Middle Ages was finally rejected. In practically all the earliest documents that have been preserved, mental disorders are described as possession (Zilboorg, 1941; Clarke, 1975; Wallis, 2010). So, when a Margery Kempe dictated her life story in 1420—which made her probably the first autobiographer in the English tradition and among women generally, quite an achievement for an illiterate countrywoman—she described her experience of what we would now term Postpartum (or postanatal) depression (PDD), but using in her explanations a rhetoric completely different than ours: devils, fiends, witches, deception, powers; and recovery through the grace, Gospel, Cross, visions of Jesus Christ (after Porter, 1991, pp. 44–46, 165–167).

The codified view followed *Malleus Maleficarum*, published in 1487 and propagating the claim that mental disorders were consequences of witchcraft and should be treated as inspired by devil and punished severely. Special attention was given to the “revelatory discovery” that the manifest source of evil were in fact women, who seduced, charmed, and possessed men, often castrating them and keeping flocks of penises as amusing independent beings (Mackay, 2009). Not long after *Malleus* was translated in English, it received royal support, as in 1597, James, still only the King of Scotland, published *Daemonologie* (James, 1597)^1^. And even in Burton's famous *The Anatomy of Melancholie*, to which I shall return several times, the influence of devils is listed among possible causes of mental disorders (Burton, 1621).

The early modern period literature about psychopathology is especially important because it introduced the idea of internal causation. It may have all started when Reginald Scott, a judge, described (Scot, 1586) many cases of witches he had tried before the court of law and claimed that these women were in fact insane. In the chapter “Not witchcraft but melancholie. Voluntarie confessions untruly made,” Scott explained that they were themselves deceived at believing they possessed supernatural powers.

In the beginning, ecclesiastical explanations were replaced by the even older notion of bodily humors. The book *De Proprietaribus Rerum* by Bartholomeus Anglicus (1240), professor of theology in Paris during the thirteenth century, was first translated in English^2^ in 1470 and published many times before the end of that century. It contained descriptions of “frenesia”—“delirium due to disease of the brain” and “perafrenesi”—“delirium occurring in the course of febrile or other systemic disease,” as well as dementia and mania, but gives best illustration of the then new approach in his depiction of depression:

Melancholy is a humour, boystous and thicke, and is bredde of troubled drastes of blode […] Of this humor havying maistry in the body, these ben the sygnes and tokens. Fyrste the colour of the skynne chaungeth into blacke or bloo: Soure savour, sharpe—and erthy is felte in the mouth. By the qualite of the humor the patient is feynte—and fereful in hert without cause, and oft sorry […] Some dread enmyte of some man: Some love and desire dethe (Chapter 11, “OfMelancholy”).

Hysteria was similarly explained in the Aristotelian manner as the movement of uterus throughout the body and its harmful effect on other organs. The term “suffocation of the mother” was widely accepted after the book of the same title by Jorden (1603). Just one token of this is King Lear's painful exclamation: “O, how this mother swells up toward my heart! *Hysterica passio* down, thou climbing sorrow; Thy element's below” (2.2.225-227; Norton Edition).

Not long after this, however, truly psychological categories were for the first time used as explanations of mental disorders. Juan Luis Vives (1492-1540), who, although Spanish by origin, lived at the court of Henry VIII, was highly influential, even considered by some to be the father of modern psychiatry (Zilboorg, 1941). Vives wrote that “The soul therefore is itself the author, the moving force (behind our functions), drawing its energy not elsewhere but within the body” (after Stone, 1998, p. 27). Just a century later, another sophisticated explanation for the conflicting nature of the mind was offered: “Therefore we must conceive in a godly man, a double selfe, one which must be denied, the other which must denie; one that breeds all the disquiet, and another that stilleth what the other hath raised…” (Sibbes, 1615, Chapter 9, “Of the souls disquiets”).

Classification of disorders was also getting more and more detailed. Robert Burton differentiated between four categories: (1) diseases emanating from the body; (2) diseases of the head (brain); (3) madness (mania); (4) melancholy. But matters very quickly grew more and more complicated. Thomas Willis, in *Cerebri Anatome*, introduced the terms neurology and psychology and wrote that disorders were caused by problems in nerve transmission, later discussing vital and involuntary systems in the brain. In the first English book on medical psychology, *De Anima Brutorum*, Willis, in 1672, described fourteen categories, including the purely neurological ones. His insights may strike us as uncannily similar to the standpoints of contemporary psychiatry, as he has described *dementia praecox* (or, in nowadays parlance—*shizophrenia*): “young persons who, lively and spirited, and at times brilliant in their childhood, passed into obtuseness and hebetude during adolescence” (Willis, 1965, p. 176); he also observed the coexistence of melancholia and mania in the same person, now known as Bipolar Affective Disorder. Willis also rejected the idea of the wandering womb as a cause of hysteria, while Sydenham (1695) wrote of hysterical convulsions that resembled epileptic seizures and, more than two centuries before Freud, was the first to discuss hysterical disorders in men, although he thought they were more prone to hypochondria. Sydenham also believed that in each case there were many sources of influence, including the family context. And even before that, Christopher Langton wrote in 1550 that sorrow can overthrow the heart and life can be utterly extinct from the patient, whom today we would label psychosomatic.

Four centuries ago, the mentally ill were equated with the lowest in the human nature, frequently even with animals, or beasts. I will draw only from the two most famous sources. Burton's *Anatomie of Melancholie* was published 10 times during the XVII century and nine more times later on, so it is considered the most widely read and quoted book in the history of psychiatry (Smith and Mulhauser, 1959). Burton introduced each type of mental disorders in verse, and here is the depiction of mania (in the chapter “Argument on the Frontispiece”):

But see the *Madman* rage down right

With furious looks, a ghastly sight.

Naked in chains bound doth he lie,

A roars amain he knows not why!

Observe him: for as in a glass,

Thine angry portraiture it was.

His picture keeps still in thy presence;

‘Twixt him and thee, there's no difference.’

Even more famous example comes from the second act of *King Lear*, where Edgar comes up with a plan to hide his identity in the following way:

Whiles I may scape

I will preserve myself, and am bethought

To take the basest and most poorest shape

That ever penury in contempt of man

Brought near to beast. My face I'll grime with filth,

Blanket my loins, elf all my hairs in knots,

And with presented nakedness outface

The winds and persecutions of the sky.

The country gives me proof and precedent

Of Bedlam beggars who with roaring voices

Strike in their numbed and mortified arms

Pins, wooden pricks, nails, springs of rosemary,

And with this horrible object from low farms,

Poor pelting villages, sheep-cotes and mills

Sometime with lunatic bans, sometime with prayers

Enforce their charity. ‘Poor Tuelygood, Poor Tom.’

(2.2.162-177; Norton Edition)

The aforementioned Bedlam was founded in 1247, but became a hospital exclusively for the mentally disturbed in 1377. For centuries, it did not offer anything close to caring and humane approach to the afflicted. It more often served for the “patients” “to be held in close confinement and totally *incommunicado*” (after Arnold, 2009, p. 25) or as a constant source of entertainment for the rich, who visited it on Sundays, bought tickets, and amused themselves by observing behavior of the psychotic. Worse still, Bedlam included a long history of corruption: in 1574, a woman was admitted without any medical cause and after that “for six weeks before her committal, she had been tied down in bed by her husband and another woman until she was ‘well nigh famished’” (Arnold, 2009, p. 42). In 1614, the first account of “malpractice on the mad” was reviewed before the College of Physicians (Arnold, 2009, p. 64), and in 1631 a surprising visit found out that Bedlam's thirty patients shared mere five pounds of cheese per day.

In the beginning of this period there was hardly anything we would consider therapeutic. The treatment recommended for witches was strangulation, beheading or burning at stake, but it was only slightly less cruel for the ill. Thomas More, who from 1516 to 1523 lived near Bedlam hospital, described the approach characteristic of his days:

“[…] he had therfor ben put uppe in bedelem, and afterward by betynge and correccyon gathered hys rememberaunce to hym, and beganne to come agayne to hym selfe beynge theruppon set at liberty and walkynge about abrode, hys olde fansyes beganne to fall agayne in his hed” More (1533, p. 198, Ch. 36).

It sounds uniquely heartless when Thomas Willis describes a case he attended: “A countrywoman, aged about 45, for long melancholic, was seized by mania […] so much so that it was necessary to bind her with chains and ropes to keep her in bed. On the fifth day half a pint of blood was drawn from the basilica vain […] In the evening I visited her. She was now shouting wildly, now singing, now weeping. She breathed rapidly, drawing the breath in with a hiss, her lips being drawn inwards. I prescribed a liniment of Vigo's ointment with [word illegible] smeared on a rose cake to be applied to her forehead and temples and a poultice of gently cooked water-hemlock to be put on the region of spleen; and also repeated drafts of a cardiac julep. The next night she died” (after Porter, 1991, p. 287).

Gradually, the prescriptions were becoming milder, but no more effective or scientific. Some treatment procedures of the time included shaving the head and washing it with rose water and vinegar in order to help evaporate “grosse vapors which hurt the Memory” (after Hunter and Macalpine, 1963, p. 23). And the mentally ill were offered herbal cures, leeching and vomiting, while no less than one Pope recommended “a roasted mouse, eaten whole” (Hunter and Macalpine, 1963, p. 12). Bright (1586) wrote that depression was a physiological disorder caused by bad diet, so that the first step in recovery was to avoid “beets, cabbage, dates, olives, bread of fine unleavened flour, pork, beef, quail, peacocks, fresh-water fish, red wine, beer and ale.” At almost the same year, a more comprehensive regime was prescribed: “Let the sicke use wyne that is white, thinne, and not very old, and let them eschewe wine that is thick and black, let there exercises be meane, let them ryde or walke by places pleasant and greene, or use sailing on water. Also a bath of sweet water with a moist dyet let the sicke use often as one of his remedies, sleep is wonderful good for them, as also moderate carnal copulation. Let them be mery as much as may be, and heare musicall instruments and singing” (Barrough, 1583, p. 36, Ch. 28, “Of Melancholie”). On his part, Robert Burton listed hundreds of herbal remedies and distracting activities (music being one of the most important among them), believing that these were effective in the cases of depression. More importantly, Burton thought that the melancholic should be encouraged to become open and confess their sorrows to an empathetic friend, thus foretelling contemporary psychotherapeutic approaches.

Similarities between contemporary psychopathology and that of the early modern England are, I believe, striking! Notions of possession and exorcism are overruled, but we are still debating the relationship between psychological and “external” factors in psychopathology. Mental disorders that we meet in our clinical practices were delineated and described about four centuries ago. Public image of the mentally ill is more affirmative then it used to be, but during the last five decades stigma has constantly been on the rise (Kecmanovic, 2010) and prevailing representation of persons with psychotic disorders is that they are dangerous and unpredictable (Link and Phelan, 1999). Asylums are still in use across Europe (Mental Health Europe, 2012) and with them discrimination, loss of human rights, torture, corruption. Our treatment approaches are not bizarre as they used to be, but their effectiveness is far from being perfect. If, however, we would like to continue improving, it may be important that we remain aware of indebtedness, past continuities, and roots of contemporary psychopathology that reach at least four-and-a-half centuries back.

## Conflict of interest statement

The author declares that the research was conducted in the absence of any commercial or financial relationships that could be construed as a potential conflict of interest.

## Footnotes

^1^It is noteworthy that when James was crowned as successor of Queen Elisabeth's throne, in 1603, he was greeted in Oxford by a theater company that performed a play about witches, Scottish king-to-be and madness of his murderous wife (see Greenblatt, 2005).

^2^Although, “Anglicus” meant “coming from England; Englishman,” he, of course, wrote in Latin.

## References

[B1] Adams J. C. (1989). Shakespeare's Physic. London: Royal Society of Medicine Press.

[B2] Anglicus B. (1240). De Proprietatibus Rerum. Early English Books Online, STC-1538-196_03.

[B3] Arnold C. (2009). Bedlam. London and Its Mad. London: Pocket Books.

[B4] Barrough P. (1583). The Methode of Phisicke Conteyning the Causes, Signes, and Cures of Invvard Diseases in Mans Body From the Head to the Foote. Early English Books Online.

[B5] Bright T. (1586). A Treatise of Melancholie. Early English Books Online, STC-3747-178_23.

[B6] Burton R. (1621). The Anatomy of Melancholy. Early English Books Online, STC-4159-951_05.

[B7] Clarke B. (1975). Mental Disorder in Earlier Britain: Exploratory Studies. Cardiff: University of Wales Press.

[B8] Edgar I. I. (1970). Shakespeare, Medicine and Psychiatry. New York, NY: Philosophical Library.

[B9] Greenblatt S. (2005). Will in the World: How Shakespeare Became Shakespeare. New York, NY: WW Norton and Company.

[B10] Hunter R. Macalpine I. (1963). Three Hundred Years of Psychiatry: 1535-1860. Oxford: Oxford University Press.

[B11] Iyengar S. (2014). Shakespeare's Medical Language: A Dictionary. London: Bloomsbury.

[B12] James I. K. (1597). Daemonologie. Early English Books Online, STC-14364-239_13.

[B13] Jorden E. (1603). A Briefe Discourse of a Disease Called the Suffocation of the Mother. Early English Books Online, STC 14790-757_26.

[B14] Kail A. C. (1986). The Medical Mind of Shakespeare. Philadelphia, PA: Williams and Wilkins.

[B15] Kecmanovic D. (2010). Can the prevention of mental illness stigma and destigmatization of people with mental illness be effectuated? Psiholoska Istrazivanja 13, 185–217.

[B16] Link B. G. Phelan J. C. (1999). The labeling theory of mental disorder (II): The consequences of labeling, in A Handbook for the Study of Mental Health. Social Contexts, Theories, and Systems, eds Horwitz A. V. Scheid T. L (Cambridge: Cambridge University Press), 361–376.

[B17] Mackay C. S. (2009). The Hammer of Witches: A Complete Translation of the Malleus Maleficarum. Cambridge: Cambridge University Press.

[B18] Mental Health Europe (2012). Mapping Exclusion. Institutional and Community-based Services in the Mental Health Field in Europe.

[B19] More T. (1533). The Apologye of Syr Thomas More, Knyght. Early English Books Online, STC-18078-137_17.

[B20] Porter R. (ed.). (1991). The Faber Book of Madness. London: Faber and Faber.

[B21] Scot R. (1586). Discoverie of Witchcraft. Early English Books Online.

[B22] Sibbes R. (1615). The Soules Conflict with it Selfe, and Victory Over it Selfe by Faith. Early English Books Online, STC-22508-666_01.

[B23] Simpson R. R. (1959). Shakespeare and Medicine. Baltimore: Williams and Wilkins.

[B24] Smith P. J. Mulhauser M. (1959). Burton's “Anatomy of Melancholy” and Burtoniana. Oxford: Oxford University Press.

[B25] Stone M. H. (1998). Healing the Mind. A History of Psychiatry from Antiquity to the Present. London: Pimlico.

[B26] Sydenham T. (1695). The Compleat Method of Curing Almost all Diseases. Early English Books Online, Wing-S6308-2241_01.

[B27] Wallis F. (ed.). (2010). Medieval Medicine: A Reader. Toronto: University of Toronto Press.

[B28] Willis T. (1965). Anatomy of the Brain and Nerves, Vol. 1 and 2. McGill-Queen's Press-MQUP. (Originally Published in Latin.)

[B29] Zilboorg G. (1941). A History of Medical Psychology. London: George Allen and Unwin.

